# Long noncoding RNA LINC00958 accelerates the proliferation and matrix degradation of the nucleus pulposus by regulating miR-203/SMAD3

**DOI:** 10.18632/aging.102436

**Published:** 2019-12-05

**Authors:** Kunchi Zhao, Yang Zhang, Hongping Yuan, Mingming Zhao, Dongxu Zhao

**Affiliations:** 1 Department of Orthopaedic Surgery, China-Japan Union Hospital of Jilin University, Changchun, Jilin 130033, P.R. China; 2 Department of Neurosurgery, The First Hospital of Jilin University, Changchun, Jilin 130021, P.R. China; 3 Department of Nephrology, Jilin FAW General Hospital, Changchun, Jilin 130011, P.R. China

**Keywords:** intervertebral disc degeneration, LINC00958, miR-203, nucleus pulposus

## Abstract

Emerging evidence suggests that long noncoding RNAs (lncRNAs) play important roles in the development of intervertebral disc degeneration (IDD). LncRNA LINC00958 has recently been shown to play crucial roles in the development of tumors. However, the role of LINC00958 in IDD remains unclear. We showed that the expression of lncRNA LINC00958 was upregulated in degenerative NP samples, and LINC00958 expression increased gradually along with the grade of exacerbation of disc degeneration. Ectopic expression of LINC00958 promoted nucleus pulposus (NP) cell proliferation, inhibited aggrecan and Col II expression and promoted MMP-2 and MMP-13 expression. In addition, we showed that miR-203 expression was downregulated in degenerative NP samples, and miR-203 expression reduced gradually along with the grade of exacerbation of disc degeneration. Moreover, we demonstrated that the expression of miR-203 was inversely related with LINC00958 expression in NP samples. Ectopic expression of miR-203 inhibited NP cell growth and inhibited ECM degradation. Furthermore, we showed that ectopic expression of miR-203 suppressed the luciferase activity of the wild-type LINC00958 3'-UTR but not the mutant LINC00958 3'-UTR. Elevated expression of LINC00958 inhibited the expression of miR-203 and promoted the expression of SMAD3. In addition, we demonstrated that lncRNA LINC00958 exerted its function by targeting miR-203 in the NP cells. These data suggested that dysregulated lncRNA LINC00958 expression might play an important role in the development of IDD.

## INTRODUCTION

Intervertebral disc degeneration (IDD) is one of the most common causes of low back pain (LBP) [1–4]. Approximately 80 percent of the population suffers from LBP at some point in their lifetime, while 10 percent of people with LBP become chronically disabled [5–7]. Although IDD is deemed to a natural process of intervertebral disc aging, several studies have demonstrated accelerated IDD due to genetic and environmental factors [1, 8–10]. IDD is characterized by the degradation of collagen, aggrecan and proteoglycans in the extracellular matrix (ECM) and nucleus pulposus (NP) cell proliferation, resulting in disrupting the homeostasis of NP and shifting intervertebral disc maintenance towards a catabolic and degenerative state [11–15]. Increasing evidence has shown that many cellular processes are involved in IDD [16–19]. However, the molecular process and mechanism of IDD remains unclear.

Long noncoding RNAs (lncRNAs) are a group of RNAs that are longer than 200 nts and that have no ability or limited ability to be coded into a protein [20–24]. Growing studies suggest that lncRNAs play crucial biological roles in diverse cellular processes including cell apoptosis, stem cell differentiation, proliferation and meiotic entry [25–29]. Moreover, emerging evidence has shown that lncRNAs were dysregulated in many tumors such as gastric cancer, hepatocellular carcinoma, osteosarcoma and lung cancer [30–33]. Recently, studies also found that lncRNAs played a critical role in the development of IDD [34–36]. For instance, Ruan et al. showed that the expression of lncRNA NEAT1 was upregulated in degenerated IVD tissues, and NEAT1 overexpression suppressed the expression of MMP13 and ADAMTS5 and induced collagen II and aggrecan expression, partly regulating the ERK/MAPK pathway. Mi et al. reported that FAF1 was overexpressed in IDD tissues, and ectopic expression of FAF1 induced NP cell growth by regulating the ERK signaling pathway. LINC00958 has recently been shown to play crucial roles in the development of tumors. For example, Seitz and colleagues first investigated the role of LINC00958 in bladder cancer [37]. They showed that LINC00958 was upregulated in bladder cancer, and knockdown of LINC00958 suppressed cell migration and viability. Guo et al. [38] reported that LINC00958 expression was upregulated in glioma cell lines and tissues, and knockdown of LINC00958 inhibited the invasion and proliferation of glioma cells by regulating miR-203 expression. Previous studies have reported that miR-203 increased the apoptosis and inflammation induced by lipopolysaccharide (LPS) by regulating NFIL3 in cardiomyocytes [39]. Given that LINC00958 and miR-203 are usually involved in the regulation of cell growth in several pathological processes and IDD is characterized by abnormal proliferation of NP cells, we supposed that LINC00958 may be overexpressed in IDD, thereby inducing NP cell growth. The major purpose of our study was to determine the role of LINC00958 in the development of IDD, the association between LINC00958 and miR-203 and their underlying mechanism in IDD.

## RESULTS

### LncRNA LINC00958 was upregulated in degenerative NP samples

**Figure 1:**
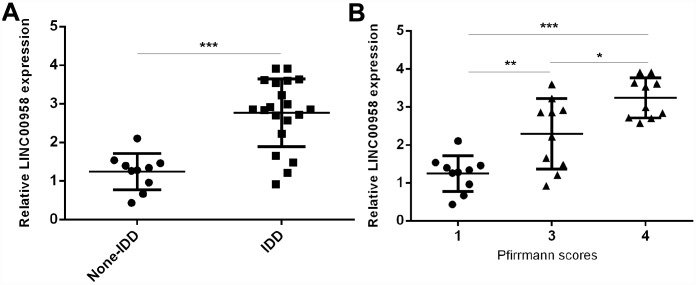
**LncRNA LINC00958 was upregulated in degenerative NP samples.** (**A**) The expression of LINC00958 was determined in 20 degenerative NP tissues and 10 scoliotic NP samples by using qRT-PCR analysis. (**B**) LINC00958 expression increased gradually along with the grade of exacerbation of disc degeneration. Data were showed as mean±SD. *p<0.05, **p<0.01 and ***p<0.001. U6 was used as the internal control.

To study the role of lncRNA LINC00958 in IDD development, we first analyzed the expression of LINC00958 in the NP tissues and the scoliotic NP samples. As shown in Figure 1A, a notably upregulated level of LINC00958 was observed in NP samples with IDD compared with the expression levels in scoliotic tissues. Furthermore, we discovered that LINC00958 expression increased gradually along with the grade of exacerbation of disc degeneration (Figure 1B).

### miR-203 expression was downregulated in degenerative NP samples

**Figure 2:**
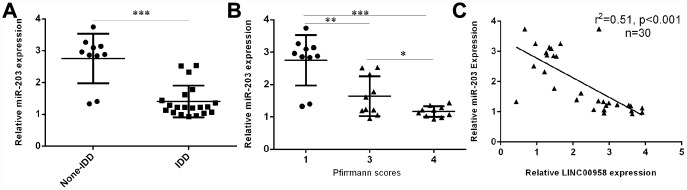
**miR-203 expression was downregulated in degenerative NP samples.** (**A**) The expression of miR-203 was determined in 20 degenerative NP tissues and 10 scoliotic NP samples by using qRT-PCR analysis. (**B**) miR-203 expression decreased gradually along with the grade of exacerbation of disc degeneration. (**C**) The expression of miR-203 was inversely related with LINC00958 expression in NP samples. *p<0.05, **p<0.01 and ***p<0.001. U6 was used as the internal control. Data were showed as mean±SD.

Next, we investigated the expression of miR-203 in the NP tissues and the scoliotic NP samples. As shown in Figure 2A, a notably downregulated level of miR-203 was observed in NP samples with IDD compared with the expression level in scoliotic tissues. Furthermore, we showed that miR-203 expression decreased gradually along with the grade of exacerbation of disc degeneration (Figure 2B). Moreover, we demonstrated that the expression of miR-203 was inversely related with LINC00958 expression in NP samples (Figure 2C).

### Interaction of lncRNA LINC00958 with miR-203

**Figure 3:**
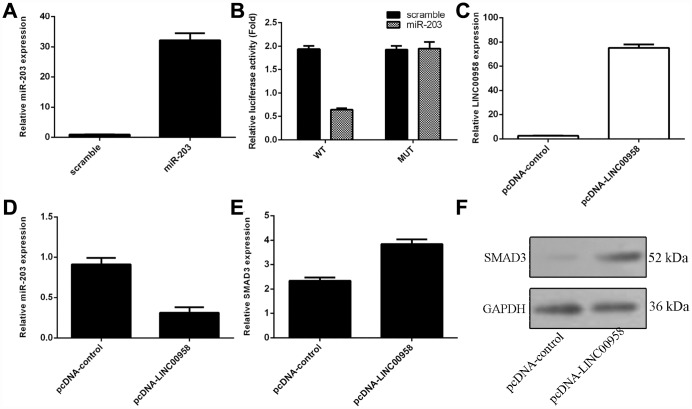
**The interaction of lncRNA LINC00958 with miR-203.** (**A**) The expression of miR-203 was measured in the NP cells by using qRT-PCR analysis. (**B**) Ectopic expression of miR-203 suppressed the luciferase activity of the wild-type LINC00958 3'-UTR but not the mutant LINC00958 3'-UTR. (**C**) The expression of LINC00958 was measured in the NP cells by using qRT-PCR analysis. (**D**) Elevated expression of LINC00958 decreased the expression of miR-203 in the NP cells. (**E**) Overexpression of LINC00958 promoted the mRNA expression of SMAD3 in the NP cells. (**F**) The protein expression of SMAD3 was detected by western blot, and GAPDH was used as the loading control. U6 was used as the internal control for miR-203. Data were showed as mean±SD.

To investigate the mechanism underlying the effect of LINC00958 on NP cell function, bioinformatics analysis was performed to find a potential target gene of LINC00958. Using qRT-PCR, we showed that overexpression of miR-203 enhanced miR-203 expression in the NP cell (Figure 3A). In addition, a dual luciferase reporter analysis indicated that ectopic expression of miR-203 suppressed the luciferase activity of the wild-type LINC00958 3'-UTR but not the mutant LINC00958 3'-UTR (Figure 3B). Moreover, the expression of LINC00958 was significantly upregulated in the NP cell after it was transfected with pcDNA-LINC00958 (Figure 3C). Elevated expression of LINC00958 decreased the expression of miR-203 in the NP cells (Figure 3D). Overexpression of LINC00958 increased the mRNA and protein expression of SMAD3 in the NP cells (Figure 3E and 3F).

### Ectopic expression of LINC00958 promoted NP cell proliferation

**Figure 4:**
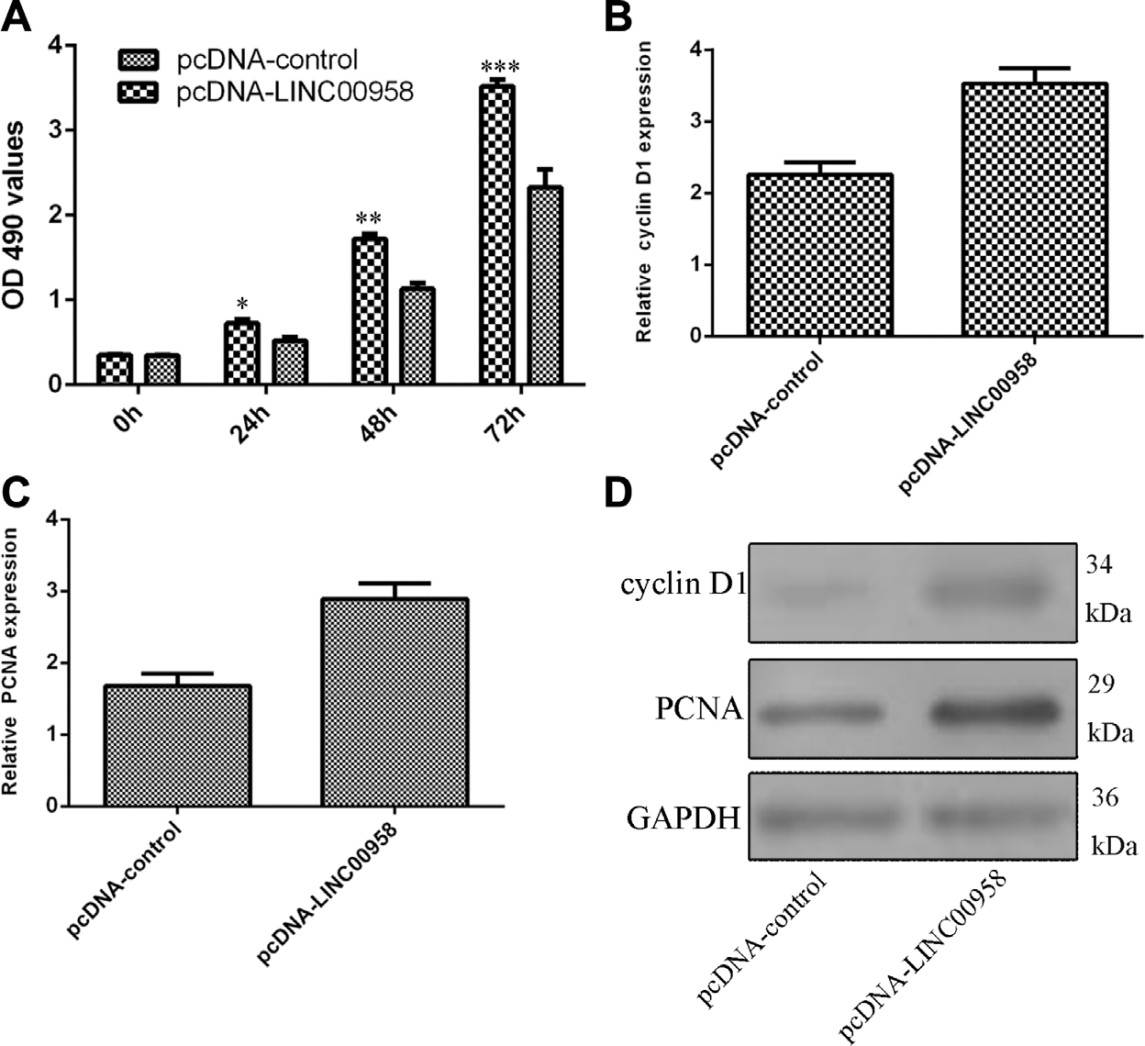
**Ectopic expression of LINC00958 promoted NP cell proliferation.** (**A**) Ectopic expression of LINC00958 promoted NP cell growth, which was determined by using the MTT assay. (**B**) Overexpression of LINC00958 increased cyclin D1 expression in the NP cell. (**C**) Elevated LINC00958 expression enhanced PCNA expression in the NP cell. (**D**) LINC00958 overexpression promoted the protein expression of cyclin D1 and PCNA. *p<0.05, **p<0.01 and ***p<0.001. GAPDH was used as the loading control. Data were showed as mean±SD.

Next, we studied the functional role of LINC00958 in the NP cells. Ectopic expression of LINC00958 promoted NP cell growth, which was determined by using the MTT assay (Figure 4A). In addition, we showed that overexpression of LINC00958 increased cyclin D1 expression in the NP cells (Figure 4B). Moreover, elevated LINC00958 expression enhanced PCNA expression in the NP cells (Figure 4C). Furthermore, we proved that LINC00958 overexpression promoted the protein expression of cyclin D1 and PCNA (Figure 4D).

### Overexpression of LINC00958 inhibited aggrecan and Col II expression and promoted MMP-2 and MMP-13 expression

**Figure 5:**
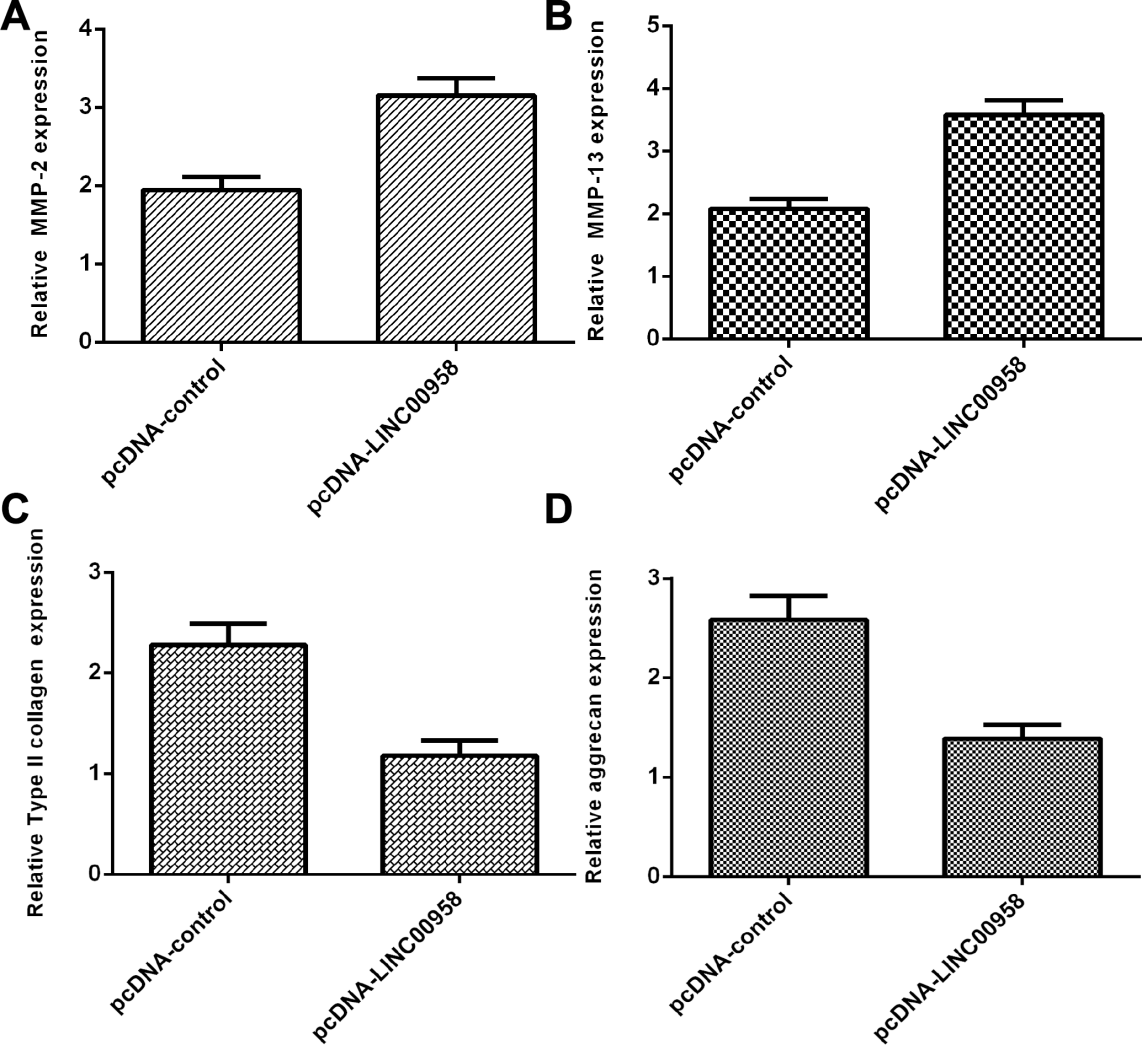
**Overexpression of LINC00958 inhibited aggrecan and Col II expression and promoted MMP-2 and MMP-13 expression.** (**A**) The expression of MMP-2 was determined by using qRT-PCR analysis. (**B**) The expression of MMP-2 was determined by using qRT-PCR analysis. (**C**) Elevated expression of LINC00958 suppressed Col II expression in the NP cells. (**D**) Overexpression of LINC00958 inhibited aggrecan expression in the NP cells. GAPDH was used as the internal control. Data were showed as mean±SD.

Elevated expression of LINC00958 increased MMP-2 expression in the NP cells (Figure 5A). In addition, we showed that LINC00958 overexpression promoted MMP-13 expression in the NP cells (Figure 5B). Moreover, we indicated that elevated expression of LINC00958 suppressed Col II expression in the NP cells (Figure 5C). Overexpression of LINC00958 inhibited aggrecan expression in the NP cells (Figure 5D).

### Ectopic expression of miR-203 decreased NP cell growth and inhibited ECM degradation

**Figure 6:**
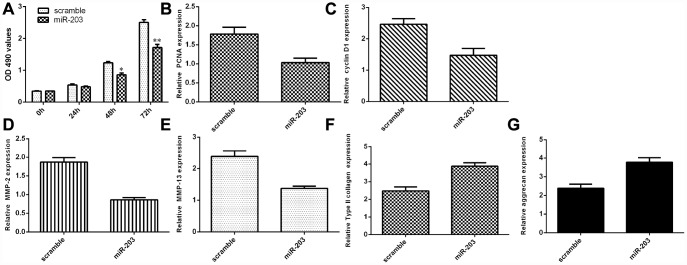
**Ectopic expression of miR-203 decreased NP cell growth and inhibited ECM degradation.** (**A**) Elevated expression of miR-203 inhibited NP cell proliferation, which was determined by MTT analysis. (**B**) The expression of PCNA was determined by using qRT-PCR analysis. (**C**) Elevated expression of miR-203 suppressed PCNA expression in the NP cells. (**D**) The expression of MMP-2 was determined by using qRT-PCR assay. (**E**) The expression of MMP-13 was determined by using qRT-PCR assay. (**F**) The expression of Col II was determined by using qRT-PCR assay. (**G**) The expression of aggrecan was determined by using qRT-PCR assay. *p<0.05, **p<0.01. GAPDH was used as the internal control. Data were showed as mean±SD.

We discovered that elevated expression of miR-203 inhibited NP cell proliferation by using MTT analysis (Figure 6A). Ectopic miR-203 expression inhibited the expression of cyclin D1 in the NP cells (Figure 6B). Elevated expression of miR-203 suppressed PCNA expression in the NP cells (Figure 6C). In addition, we showed that overexpression of miR-203 suppressed the expression of MMP-2 and MMP-13 in the NP cells (Figure 6D and 6E). Moreover, elevated expression of miR-203 induced the expression of Col II and aggrecan in the NP cells (Figure 6F and 6G).

### LncRNA LINC00958 exerted its function by targeting miR-203 in the NP cells

**Figure 7:**
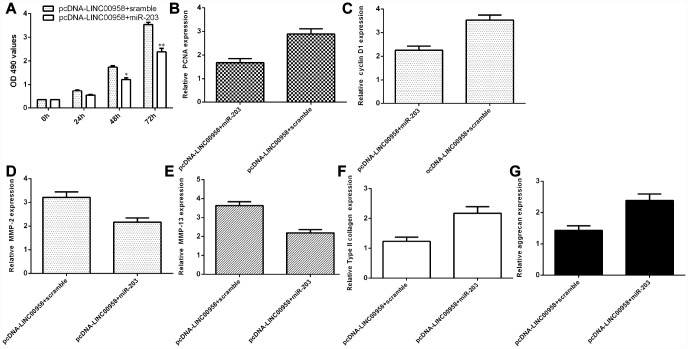
**LncRNA LINC00958 exerted its function by targeting miR-203 in the NP cells.** (**A**) Cell proliferation was measured by using the MTT assay. (**B**) PCNA expression was determined by qRT-PCR. (**C**) The expression of cyclin D1 was measured by qRT-PCR. (**D**) Elevated expression of LINC00958 suppressed the expression of MMP-3 in the LINC00958-overexpressing NP cells. (**E**) The expression of MMP-13 was determined by qRT-PCR analysis. (**F**) Col II expression was measured by using qRT-PCR analysis. (**G**) The expression of aggrecan was detected by using qRT-PCR analysis. *p<0.05, **p<0.01. GAPDH was used as the internal control. Data were showed as mean±SD.

To determine whether the functional effect of LINC00958 in promoting NP proliferation and ECM degradation was due to targeting miR-203, miR-203 was overexpressed in the NP cells through transfection with miR-203 mimic and pcDNA-LINC00958. The MTT analysis indicated that ectopic expression of miR-203 inhibited cell proliferation in the LINC00958-overexpressing NP cells (Figure 7A). In line with this result, the qRT-PCR assay showed that miR-203 overexpression suppressed cyclin D1 (Figure 7B) and PCNA (Figure 7C) expression in the LINC00958-overexpressing NP cells. Furthermore, the qRT-PCR assay demonstrated that elevated expression of LINC00958 suppressed MMP-3 (Figure 7D) and MMP-13 (Figure 7E) expression and enhanced Col II (Figure 7F) and aggrecan (Figure 7G) expression in the LINC00958-overexpressing NP cells.

## DISCUSSION

In our study, we showed that the expression of lncRNA LINC00958 was upregulated in degenerative NP samples, and LINC00958 expression increased gradually along with the grade of exacerbation of disc degeneration. Ectopic expression of LINC00958 promoted NP cell proliferation, inhibited aggrecan and Col II expression and promoted MMP-2 and MMP-13 expression. In addition, we showed that miR-203 expression was downregulated in degenerative NP samples, and miR-203 expression decreased gradually along with the grade of exacerbation of disc degeneration. Moreover, we demonstrated that the expression of miR-203 was inversely correlated with LINC00958 expression in NP samples. Ectopic expression of miR-203 inhibited NP cell growth and inhibited ECM degradation. Furthermore, we showed that ectopic expression of miR-203 suppressed the luciferase activity of the wild-type LINC00958 3'-UTR but not the mutant LINC00958 3'-UTR. Elevated expression of LINC00958 inhibited the expression of miR-203 and promoted the expression of SMAD3. In addition, we demonstrated that lncRNA LINC00958 exerted its function by targeting miR-203 in the NP cells. These data suggested that dysregulated lncRNA LINC00958 expression might play an important role in the development of IDD.

LINC00958 has been shown to play important roles in the development of tumors. For example, Seitz and colleagues investigated the role of LINC00958 in bladder cancer [37]. They used RNA-sequencing to find aberrantly expressed lncRNAs in bladder cancer. Their data suggested that LINC00958 was upregulated in bladder cancer and knockdown of LINC00958 suppressed cell migration and viability. Silencing of LINC00958 affected resistance to anoikis and invasion. Chen and colleagues showed that LINC00958 was overexpressed in endometrial cancer, and LINC01480 was shown to be required to express the micropeptide [40]. He and colleagues demonstrated that LINC00958 was upregulated in bladder tumor-associated lymphatic metastasis and lymphangiogenesis [41]. Ectopic expression of LINC00958 promoted VEGF-C expression by associating with WDR5. However, the expression and functional role of LINC00958 remained unknown. In our study, we first determined the expression of LINC00958 in the degenerative NP samples. We found that LINC00958 expression was upregulated in degenerative NP samples, and LINC00958 expression increased gradually along with the grade of exacerbation of disc degeneration. It is suggested that there is correlation between expression of LINC00958 and low back pain. In addition, we showed that LINC00958 overexpression induced NP cell proliferation, decreased aggrecan and Col II expression and promoted MMP-2 and MMP-13 expression.

Furthermore, we analyzed the potential mechanism of LINC00958 in the development of IDD. Previous studies have suggested that lncRNAs act as a molecular sponge of miRNA, modulating its expression [42, 43]. In addition, Guo et al. [38] reported that LINC00958 expression was upregulated in glioma cell lines and tissues, and knockdown of LINC00958 inhibited glioma cell invasion and proliferation by regulating miR-203 expression. Nevertheless, the relationship between LINC00958 and miR-203 in the function of NP cells remains to be studied. In our study, we demonstrated that ectopic expression of LINC00958 decreased the expression of miR-203 in NP cells. In addition, we demonstrated that miR-203 expression was downregulated in degenerative NP samples, and miR-203 expression decreased gradually along with the grade of exacerbation of disc degeneration. Moreover, we demonstrated that the expression of miR-203 was inversely related with LINC00958 expression in NP samples. Hu et al. [44] reported that miR-203 prevented the deposition and synthesis of ECM components via a SMAD3-dependent mechanism. In line with this finding, we demonstrated that ectopic expression of miR-203 inhibited NP cell growth and ECM degradation. Finally, we demonstrated that LncRNA LINC00958 exerted its function by targeting miR-203 in the NP cells.

## Limitation

The major limitation of our research was that the control NP tissues are from scoliosis patients [45, 46]. Healthy age and spine level matched cadaveric discs were better to use as the control NP tissues. However, it is difficult to obtain the healthy age and spine level matched cadaveric discs.

In summary, we demonstrated that LINC00958 expression was upregulated in degenerative NP samples, and LINC00958 expression increased gradually along with the grade of exacerbation of disc degeneration. Ectopic expression of LINC00958 promoted NP cell proliferation, inhibited aggrecan and Col II expression and promoted MMP-2 and MMP-13 expression partly by regulating miR-203 expression. These results suggested that dysregulated LINC00958 expression may play an important role in the development of IDD.

## MATERIALS AND METHODS

### Tissues

Human lumbar disc tissues were obtained from patients with IDD (n =20; average age 43 ± 7.4 years, range 31–56 years) or scoliosis (n= 10; average age 20 ± 1.2 years, range 18–22 years) who were undergoing spinal fusion at the China-Japan Union Hospital of Jilin University (Jilin, China). The tissues of the scoliosis patients were used as the control. These samples were immediately frozen in liquid nitrogen until they were used. The degree of disc degeneration was determined by a magnetic resonance imaging (MRI) scan following the modified Pfirrmann classification (Supplementary Table 1). This study was approved by the ethics committee of Jilin University. Written informed consent was collected from each patient.

### Nucleus pulposus cell culture and transfection

Human nucleus pulposus (NP) cells were isolated and cultured following the procedures outlined in several previous studies [11–13]. NP cells were cultured in Dulbecco’s modified Eagle’s medium (DMEM)/F12 containing fetal bovine serum (FBS), streptomycin, penicillin and l-glutamine. pcDNA-control, pcDNA-LINC00958, miR-203 mimic and scramble were obtained from GenePharma (Shanghai, China). Cell transfection was performed by using Lipofectamine 3000 (Invitrogen, CA, USA) according to the manufacturer’s instructions.

### RNA isolation and qRT-PCR

Total RNA was isolated from NP cells or samples with TRIzol (Invitrogen, CA, USA). The concentration of RNA was detected using a NanoDrop spectrophotometer (NanoDrop Technologies, DE, USA). cDNA (First-strand complementary DNA) synthesis was performed from 1 mg of RNA in a 12 ml volume containing dNTP Mix (Invitrogen, USA) and stem-loop primer. The mix was then incubated for 5 min at 65 °C and mixed with 0.1 M DTT, 5xRT buffer, RNase inhibitor and MultiScribe reverse transcriptase (Invitrogen, USA). Real-time quantitative polymerase chain reactions (qRT-PCRs) were performed to measure the expression of miRNA, lncRNA and mRNA using the SYBR Green PCR kit on the 7000 Sequence Detection System (Applied Biosystems, CA, USA). GAPDH and U6 were used as the internal controls for mRNA, lncRNA, and miRNA expression. The relative expression was determined by the 2^−ΔΔCt^ method. The primer sequences used in this study were as follows: MMP-2, (forward) 5ʹ-GGCCCTGTCACTCCTGAG AT-3ʹ and (reverse) 5ʹ-GGCATCCAGGTTATCGGG GA-3ʹ; GADPH, (forward) 5ʹ-GGCCTCCAAGGAGT AAGACC-3ʹ and (reverse) 5ʹ-AGGGGTCTACAT GGCAACTG-3ʹ; SMAD3 (forward) 5ʹ-CGATGTCC CCAGCACACAATAAC-3ʹ and (reverse) 5ʹ-TAGTA GGAGATGGAGCACCAAAAGG-3ʹ; miR-203 (forward) 5ʹ- CGATGCTGTGAAATGTTTAGGGAC-3ʹ and (reverse) 5ʹ-TATGGTTTTGACGACTGTGT GAT-3ʹ; U6 (forward) 5ʹ-ATTGGAACGATACAG AGAAGATT-3ʹ and (reverse) 5ʹ-GGAACGCTTCACG AATTTG-3ʹ; and LINC00958, (forward) 5ʹ-CC ATTGAAGATACCACGCTGC-3ʹ and (reverse) 5ʹ-G GTTGTTGCCCAGGGTAGTG-3ʹ.

### Western blotting

Total protein was isolated from NP cells or samples with RIPA buffer (Sigma, MO, USA). A total of 20 μg of protein was separated with 10% SDS-PAGE (sodium dodecyl sulphate-polyacrylamide) and transferred to membranes (Millipore, MA, USA). The membrane was incubated with the primary antibodies (SMAD3, catalogue: SAB4504210 and GAPDH, catalogue: G9545, Sigma; PCNA, catalogue: SAB2502098 and cyclin D1, catalogue: SAB1306586) at 4 °C overnight. Then, the membrane was labeled with horseradish peroxidase (HRP)-conjugated anti-IgG antibody. The protein signal was measured with the ECL system. GAPDH was used as the internal control.

### Cell proliferation assays

Cell growth was evaluated by 3-(4,5-dimethyl-2-thiazolyl)-2,5-diphenyl-2-H-tetrazolium bromide (MTT) according to manufacturer’s information. Cells were cultured in a 96-well plate, and cell proliferation was measured at different time points. A total of 20 μl of MTT solution was added to each well, and they were cultured for an additional three hours. Then, the absorbance was determined at 490 nm using a Spectra microplate reader (Molecular Devices, CA, USA).

### Luciferase reporter assay

Bioinformatics databases (http://starbase.sysu.edu.cn/index.php) were used to predict binding sequences between miR-203 and LINC00958. miR-203 was predicted to have putative binding sites of LINC00958. The seed sequences from LINC00958 and its mutant were subcloned into the downstream luciferase gene in the Dual-luciferase Expression Vector (Promega, WI). For the luciferase reporter analysis, cells were cultured in a 96-well plate and cotransfected with pmirGLO-LINC00958-wt or pmirGLO-LINC00958-mut and miR-203 mimic or scramble by the Lipofectamine 3000 reagent (Invitrogen, USA). The luciferase activity was detected by using the Dual-Luciferase Assay System (Promega).

### Statistical analysis

Data are shown as the mean ± standard deviation (SD). Statistical analysis was performed using SPSS 18.0 (SPSS, IL, USA). The significant differences between groups were determined by analysis of variance or Student’s t test. A P value < 0.05 was considered statistically significant.

## Supplementary Material

Supplementary Table 1
