# Supplementary material for: Long noncoding RNA LINC00958 accelerates the proliferation and matrix degradation of the nucleus pulposus by regulating miR-203/SMAD3
Source: Aging (Albany NY). 2019 Dec 5;11(23):10814–25. doi: 10.18632/aging.102436 (PMC6932897; doi:10.18632/aging.102436)
Supplement: Supplementary Table 1 [file aging-11-102436-s001..pdf]

SUPPLEMENTARY TABLE

Supplementary Table 1. Clinical Finding in 20 Patients with degenerated disc.

Patient No.	Sex/Age (yr)	Type of LDH	Level	Duration of Symptoms(mo)
1	F/51	P	L4/L5	13
2	M/41	P	L4/L5	11
3	F/35	SE	L5/S1	4
4	F/31	TE	L4/L5	16
5	M/32	S	L3/L4	8
6	F/34	S	L3/L4	7
7	M/52	TE	L4/L5	5
8	F/45	TE	L4/L5	6
9	M/44	SE	L4/L5	31
10	F/32	TE	L5/S1	21
11	M/50	S	L5/S1	4
12	F/35	TE	L4/L5	13
13	M/53	S	L3/L4	7
14	F/52	P	L3/L4	1
15	M/41	TE	L5/S1	11
16	F/33	P	L5/S1	13
17	M/46	S	L4/L5	12
18	M/56	SE	L4/L5	4
19	F/42	SE	L4/L5	12
20	F/55	P	L3/L4	7

F, indicates female; M, male; P, protrusion; S, sequestration; SE, subligamentous extrusion; TE, transligamentous extrusion; L, lumbar; S, sacral; LDH: lumbar disc herniation.
